# Functional Connectivity Alterations between Networks and Associations with Infant Immune Health within Networks in HIV Infected Children on Early Treatment: A Study at 7 Years

**DOI:** 10.3389/fnhum.2017.00635

**Published:** 2018-01-11

**Authors:** Jadrana T. F. Toich, Paul A. Taylor, Martha J. Holmes, Suril Gohel, Mark F. Cotton, Els Dobbels, Barbara Laughton, Francesca Little, Andre J. W. van der Kouwe, Bharat Biswal, Ernesta M. Meintjes

**Affiliations:** ^1^MRC/UCT Medical Imaging Research Unit, Division of Biomedical Engineering, Department of Human Biology, Faculty of Health Sciences, University of Cape Town, Cape Town, South Africa; ^2^African Institute for Mathematical Sciences, Muizenberg, South Africa; ^3^Scientific and Statistical Computing Core, National Institutes of Health, Bethesda, MD, United States; ^4^Department of Health Informatics, School of Health Professions, Rutgers University, Newark, NJ, United States; ^5^Family Clinical Research Unit, Department of Paediatrics and Child Health, Stellenbosch University, Stellenbosch, South Africa; ^6^Department of Statistical Sciences, University of Cape Town, Cape Town, South Africa; ^7^Department of Radiology, Massachusetts General Hospital, Boston, MA, United States; ^8^Department of Biomedical Engineering, New Jersey Institute of Technology, Newark, NJ, United States

**Keywords:** HIV infection, fMRI, functional connectivity, resting state networks, seed-based correlation analysis, children, neurodevelopment, CD4

## Abstract

Although HIV has been shown to impact brain connectivity in adults and youth, it is not yet known to what extent long-term early antiretroviral therapy (ART) may alter these effects, especially during rapid brain development in early childhood. Using both independent component analysis (ICA) and seed-based correlation analysis (SCA), we examine the effects of HIV infection in conjunction with early ART on resting state functional connectivity (FC) in 7 year old children. HIV infected (HIV+) children were from the Children with HIV Early Antiretroviral Therapy (CHER) trial and all initiated ART before 18 months; uninfected children were recruited from an interlinking vaccine trial. To better understand the effects of current and early immune health on the developing brain, we also investigated among HIV+ children the association of FC at 7 years with CD4 count and CD4%, both in infancy (6–8 weeks) and at scan. Although we found no differences within any ICA-generated resting state networks (RSNs) between HIV+ and uninfected children (27 HIV+, 18 uninfected), whole brain connectivity to seeds located at RSN connectivity peaks revealed several loci of FC differences, predominantly from seeds in midline regions (posterior cingulate cortex, paracentral lobule, cuneus, and anterior cingulate). Reduced long-range connectivity and increased short-range connectivity suggest developmental delay. Within the HIV+ children, clinical measures at age 7 years were not associated with FC values in any of the RSNs; however, poor immune health during infancy was associated with localized FC increases in the somatosensory, salience and basal ganglia networks. Together these findings suggest that HIV may affect brain development from its earliest stages and persist into childhood, despite early ART.

## Introduction

Increased access to antiretroviral therapy (ART) has transformed human immunodeficiency virus (HIV) infection from a fatal to a chronic illness. However, unlike HIV, many antiretrovirals (ARVs) do not effectively penetrate the blood-brain barrier of the central nervous system (CNS), so that the brain becomes a sanctuary site for HIV resulting in long-term damage and delayed neurodevelopment (see for example Martin et al., 2006; Smith et al., 2006; van Rie et al., 2009; Laughton et al., 2013; van Arnhem et al., 2013; Whitehead et al., 2014).

Even in the ART era, HIV infected (HIV+) children demonstrate cognitive delay and motor deficits compared to uninfected controls, along with impaired language abilities, failure to reach developmental milestones (Martin et al., 2006; van Rie et al., 2006; Koekkoek et al., 2008; Laughton et al., 2013; van Arnhem et al., 2013), and behavioral problems (Govender et al., 2011; Musielak and Fine, 2016), demonstrating the ongoing influence of the virus on the developing brain on ART.

Neuroimaging allows direct examination of how the pediatric brain is altered in the presence of both HIV and its treatment. Previous findings in HIV+ children on ART include ventricular enlargement, white matter (WM) abnormalities, cortical and subcortical volume alterations, and calcification of the basal ganglia and corpus callosum (Sarma et al., 2013; van Arnhem et al., 2013; Hoare et al., 2014; Uban et al., 2015; Cohen et al., 2016; Lewis-de los Angeles et al., 2016, 2017; Yadav et al., 2017). Within these studies, clinical, immunologic, and virologic measures were associated with volumetric measures, WM alterations, diffusivity markers, and shape deformation (van Arnhem et al., 2013; Uban et al., 2015; Cohen et al., 2016; Lewis-de los Angeles et al., 2016). Since HIV penetrates the CNS during the first 3 weeks of life of perinatally HIV+ children (González-Scarano and Martín-García, 2005), which corresponds to a critical period in development, markers of early immune health, or virologic status may play an integral part in determining later neurological outcomes (Bilbo, 2013). Notably, children in these earlier studies initiated ART at different ages, mostly after 2 years of age, and often with limited viral load (VL) suppression. Earlier ART initiation and VL suppression could potentially prevent or reduce these HIV-related brain changes.

Following the landmark Children with HIV Early Antiretroviral Therapy (CHER) trial (Violari et al., 2008; Cotton et al., 2013) showing reduced infant mortality and HIV progression in infants initiating ART below 12 weeks of age compared to standard 2006 guidelines that advised initiating ART when CD4 lymphocyte percentage (CD4%) declined below 25% or for severe clinical disease (WHO, 2006; Violari et al., 2008; Cotton et al., 2013; Laughton et al., 2014), all guidelines now recommend initiating ART as soon as possible for all HIV+ infants regardless of CD4 measures, even if asymptomatic (WHO, 2013). Although early ART improves neurodevelopmental outcomes (Laughton et al., 2013; Brahmbhatt et al., 2014; Crowell et al., 2015), the long-term effects of early lifelong ART on brain development has not been established. We have found, for example, that alterations in brain WM and basal ganglia metabolism are evident at age 5 years in children from the CHER cohort despite starting ART before 75 weeks of age and VL suppression (Ackermann et al., 2016; Mbugua et al., 2016). In addition, there is concern about possible adverse effects of long-term ART including metabolic abnormalities (Vigano et al., 2010) and neurotoxicity (Robertson et al., 2012).

Resting state functional magnetic resonance imaging (RS-fMRI) provides unique information regarding the functional connectivity (FC) of spatially distinct brain regions and the integrity of intrinsic resting state brain networks (RSNs). Since brain activity is measured when subjects are not performing a specific task, it greatly reduces the potentially confounding influences of attention, task performance, and language comprehension and is ideally suited to pediatric studies. It is a sensitive marker of alterations in brain development (Superkar et al., 2010; Thomason et al., 2011; de Bie et al., 2012) and disease (Greicius, 2008).

In HIV+ adults, RS-fMRI studies show reduced FC within various brain networks, including the visual (Wang et al., 2011), default mode (DM), executive control and salience networks (Thomas et al., 2013), as well as HIV-related changes in integration within the DM and executive control networks (Thomas et al., 2015), attenuated frontostriatal connectivity (Ipser et al., 2015), and both decreases (DM to dorsal attention, DM to salience, executive control to sensorimotor) and increases (executive control to salience) in internetwork correlations (Thomas et al., 2013). Conversely, Ortega et al. (2015) found similar FC within the DM network (DMN) in patients on ART and uninfected controls, and higher FC within the ventral attention network in patients on ART than those not receiving ART, suggesting that ART may mitigate HIV-related FC alterations. Notably, partial correlations between subcortical seeds revealed no changes in subcortical connectivity in HIV+ adults on long-term ART with at least 1 year of undetectable plasma HIV ribonucleic acid (RNA) compared to uninfected controls (Janssen et al., 2017). The only RS-fMRI study performed to date in HIV+ youth, all of whom were on ART, showed associations of disease severity, characterized by higher peak HIV RNA and lower nadir CD4%, with poorer FC within the DMN, as well as decreases and increases in connectivity of seeds within the DMN to regions in the executive control, sensorimotor, salience, anterior cingulate/precuneus and visual networks (Herting et al., 2015). Peak plasma HIV RNA and nadir CD4% reflect the worst virologic status and immune health of subjects, respectively. The finding of lower within- and greater between-network connectivity, a pattern of connectivity that occurs earlier in development (Fair et al., 2007, 2009; Power et al., 2010), suggests developmental delay in youths with more advanced disease severity.

Here we use RS-fMRI to examine FC differences at age 7 years in HIV+ children from the CHER cohort compared to uninfected controls and, among infected children, associations of FC with measures of immune health. All HIV+ children initiated ART before 18 months of age and were virologically suppressed at the time of scanning. Major strengths of this study include close monitoring since birth, standardized ART regimens, recruitment from similar socio-demographic and economic backgrounds, and scanning within 6 months of their 7th birthdays. First, we hypothesized that, compared to uninfected children, HIV+ children would show reduced FC within and between the DM, executive control, somatosensory, salience and visual networks. Second, we postulated that improved immune health, measured by CD4 count and percentage in infancy and at scan, would be related to greater functional connectivity in these networks.

## Methods

### Participants

Participants were 38 HIV+ Xhosa children (mean age ± standard deviation = 7.22 ± 0.16 years; 17 males) from the randomized CHER trial in follow-up at the Family Clinical Research Unit, Tygerberg Children's Hospital, in Cape Town, South Africa (Violari et al., 2008; Cotton et al., 2013) and 29 uninfected children (7.17 ± 0.10 years; 14 males) from an interlinking vaccine trial (Madhi et al., 2010). The two studies in parallel recruited infected (CHER) and uninfected (vaccine trial) infants from the same community in Cape Town. Inclusion criteria for both studies included birth weight >2,000 g and no CNS problems (other than due to HIV) or dysmorphic syndromes. A summary description of socioeconomic data from a subset of the cohort is published elsewhere (Holmes et al., 2017); although that study only included uninfected children, the data are representative of the community.

In the CHER trial, HIV+ infants 6–12 weeks of age with CD4% ≥25% were randomized to one of three treatment regimens: limited ART for either 40 or 96 weeks and restart when clinical and/or immunological criteria were met, or to start ART only if they became symptomatic or CD4% dropped below 20% (25% in the first year; Violari et al., 2008; Cotton et al., 2013), as per guidelines at the time (WHO, 2006). All HIV+ children had started ART before 18 months of age and received comprehensive immunological and clinical follow-up thereafter as described previously (Violari et al., 2008; Cotton et al., 2013). First line ART regimen consisted of Zidovudine (ZDV) + Lamivudine (3TC) + Lopinavir-Ritonavir (LPV/r, Kaletra) (Violari et al., 2008; Cotton et al., 2013). Children born to HIV+ mothers were exposed to treatment for prevention of mother-to-child transmission (PMTCT), mostly Zidovudine antenatally from 28 to 34 weeks and a single dose of Nevirapine (NVP) to the mother and Zidovudine for a week and a single dose of NVP to the infant. Of the 18 uninfected children, 8 were born to HIV+ mothers. Other than this single dose given to exposed infants as part of PMTCT, uninfected children never received ART.

### MRI acquisition

Children were scanned on a 3T Allegra MRI (Siemens, Erlangen, Germany) at the Cape Universities Brain Imaging Centre (CUBIC) in Cape Town, South Africa, according to protocols approved by the Faculty of Health Sciences Human Research Ethics Committees of both the Universities of Cape Town and Stellenbosch. All parents and guardians provided written informed consent and all children provided oral assent.

T1-weighted structural images were acquired in the sagittal plane using a motion navigated (Tisdall et al., 2009) multi echo magnetization prepared rapid gradient echo (MEMPRAGE) sequence (van der Kouwe et al., 2008) with TR 2,530 ms, TEs 1.53/3.19/4.86/6.53 ms, inversion time (TI) 1,160 ms, flip angle 7°, resolution 1.3 × 1 × 1 mm^3^, and field of view (FOV) 224 × 224 × 144 mm^3^. RS-fMRI data were acquired using an interleaved multi-slice 2D gradient echo, echo planar imaging (EPI) sequence: 33 interleaved slices, slice thickness 4 mm, slice gap 1 mm, voxel size 3.44 × 3.44 × 5 mm^3^, FOV 220 × 220 × 164 mm^3^, TR/TE 2,000/30 ms, flip angle 77°, 180 volumes.

### RS-fMRI processing

RS-fMRI data were preprocessed in AFNI (Cox, 1996) with a pipeline specified using the afni_proc.py tool (see Appendix A for details). Briefly, preprocessing included: removal of the first 5 TRs; despiking; slice timing alignment; alignment to the skull-stripped structural image and nonlinear warping to 3 mm Talairach-Tournoux (TT) standard space; volume registration using 6 degrees of freedom (DOF); spatial smoothing with a Gaussian kernel of 6 mm full width at half maximum (FWHM); segmentation of the structural image into WM, gray matter (GM) and cerebrospinal fluid (CSF), and regression of the eroded WM and CSF average time series along with their derivatives; and bandpass filtering between 0.01–0.1 Hz as low frequency fluctuation (LFF) interval. Subjects were excluded if their structural or RS-fMRI data sets were of a poor image quality, contained signal dropout, or significant artifacts, or could not be aligned to the standard template. Time series were truncated to exclude suprathreshold subject motion, defined as >3 mm translation or >3 degrees rotation in any direction. Subjects with fewer than 130 time points after truncation were excluded and the time series of all remaining subjects were reduced to 130 time points to maintain equal weightings per subject.

A single, representative motion parameter was also estimated for each subject for inclusion as a control variable in the model design of the main analyses. First, the framewise displacement ($\text{F}{\text{D}}_{i}=\sqrt{{\left(x_{i}-x_{i-1}\right)}^{2}+{\left(y_{i}-y_{i-1}\right)}^{2}+{\left(z_{i}-z_{i-1}\right)}^{2}}$) (Yan et al., 2013) was calculated with an in-house script for each volume relative to the previous volume using the translation parameters computed during motion correction. Then, *FD*_*i*_ values were averaged for each participant to estimate the time series mean framewise displacement (FD). Two sample *t*-tests were used to compare FD values between the HIV+ and uninfected groups.

Group analyses were performed using tools within AFNI (Cox, 1996), FSL (Smith et al., 2004) and in-house scripts. Group independent component analysis (ICA) was performed to define RSNs and locations of peak FC within each network, which were subsequently used as seeds in our seed-based correlation analysis (SCA). SCA was performed to study whole brain (WB) connectivity to the areas of peak network connectivity. While ICA is useful for examining functional connectivity within networks, SCA generates seed-to-WB connectivity maps and permits an examination of FC differences that may occur between networks (as well as within networks, without the ICA-based condition of spatial independence of components).

### ICA-generated RSNs

Standard RSNs were identified using group ICA with FSL's MELODIC function. Twenty independent components (ICs) were generated from the complete set of processed time series (i.e., from all subjects, after any exclusion criterion from quality control, etc. were applied), based on standard dimensionality reduction used in RS-fMRI studies of similar group size (Smith et al., 2009). Each IC was visually inspected and quantitatively compared to the standard set of Functional Connectome Project (FCP) template RSN maps (Biswal et al., 2010) using the 3dMatch function in FATCAT (Taylor and Saad, 2013). ICs containing known networks were thresholded at *Z* > 3 and binarized RSN masks created. The remaining ICs (representing non-GM tissue, subject motion, etc.) were discarded. The FSL function dual_regression (Beckmann et al., 2009) was also used to generate FC maps (Z-scores) associated with each RSN for each individual.

### SCA-generated WB FC maps

For SCA, spherical seeds of 5 mm radius, constrained by the ICA-generated RSN masks, were placed at the global peak of each ICA-generated RSN. In RSNs with large anteroposterior (AP) spread, a second seed was placed at a distant local maximum along the AP direction to explore potentially varied features of the network. Additional seeds were not selected in predominantly lateral networks, as left-right homotopy tends to be reflected in high temporal correlation along the left-right axis. The average time series of each seed was correlated with that of every voxel in the WB. The Pearson *r*-values from SCA were Fisher Z-transformed to generate a WB FC map for each seed for each subject.

### Statistical analyses

FC in HIV+ and uninfected children were compared both within ICA-generated RSNs and SCA-generated WB FC maps using voxelwise two sample, unpaired *t*-tests with FSL *randomize* (Winkler et al., 2014). Among infected children, we also used FSL *randomize* to examine associations of FC (from dual_regression) within the ICA-generated RSNs with measures of immune health (CD4 and CD4%) both at infancy and time of scan. Subject sex and FD were included in the model as confounding variables; subject age was not included due to the narrow age range of participants.

The significance of clusters was determined with AFNI's 3dClustSim using mixed autocorrelation function (ACF) modeling to account for the spatial smoothness of noise (Cox et al., 2017) at a voxelwise significance threshold of *p* = 0.005 and clusterwise significance of α < 0.05 (with 5,000 Monte Carlo simulations).

## Results

Of 38 HIV+ and 29 controls, 9 (5 HIV+) were excluded due to significant ghosting artifacts or poor image quality, and 13 (6 HIV+) due to not meeting motion criteria. Therefore, our final sample included 27 HIV+ (18 female) and 18 uninfected (11 female) children (Table 1). Groups did not differ in age, sex, birth weight, or FD during scanning. Children initiated ART at a median age of 10 weeks (IQR: 8–23), and were all still on first line ART with plasma HIV RNA below detectable limits at time of scanning. Due to early ART, VLs were suppressed at a young age in all children—by 12 months in 81% of children, and by 2 years in 96% of children.

**Table 1:** Sample characteristics.

	**HIV uninfected**	**HIV infected**
**DEMOGRAPHICS**		
N	18	27
Age (years)	7.2 ± 0.2	7.2 ± 0.1
Sex (%M)	39%	33%
Birth weight (*g*)	3,079 ± 493	3,077 ± 528
Motion (mm)^a^	0.13 ± 0.09	0.12 ± 0.07
**TREATMENT-RELATED MEASURES**		
Age of ART initiation (weeks)		10 (8–23)
Time on ART treatment (weeks)		335 ± 40
Children on interrupted ART^b^		12 (44%)
Age of interruption (weeks)		63 ± 26
Duration of interruption (weeks)		36 (24–54)
**CLINICAL DATA AT ENROLLMENT**		
CD4 count		1,936 ± 768
CD4%		34 ± 9
CD8^b^		1,606 ± 812
CD4/CD8^c^		1.5 ± 0.9
Plasma Viral loads (RNA copies/ml)
High (>750,000)		11 (40%)
Low (400–750,000)		16 (59%)
Suppressed (< 400)		0
**CLINICAL DATA AT SCAN**		
CD4 count		1,222 ± 400
CD4%		36 ± 6
Plasma Viral loads (RNA copies/ml)
High (>750,000)		0
Low (400–750,000)		0
Suppressed (< 400)		27 (100%)

*Values are Mean ± Standard Deviation or Median (Interquartile range)*.

a *Motion assessed using Framewise Displacement*.

b *9 children were interrupted around 40 weeks of age, and 3 children around 96 weeks*.

c *CD8 missing for one child*.

**Figure 1:**
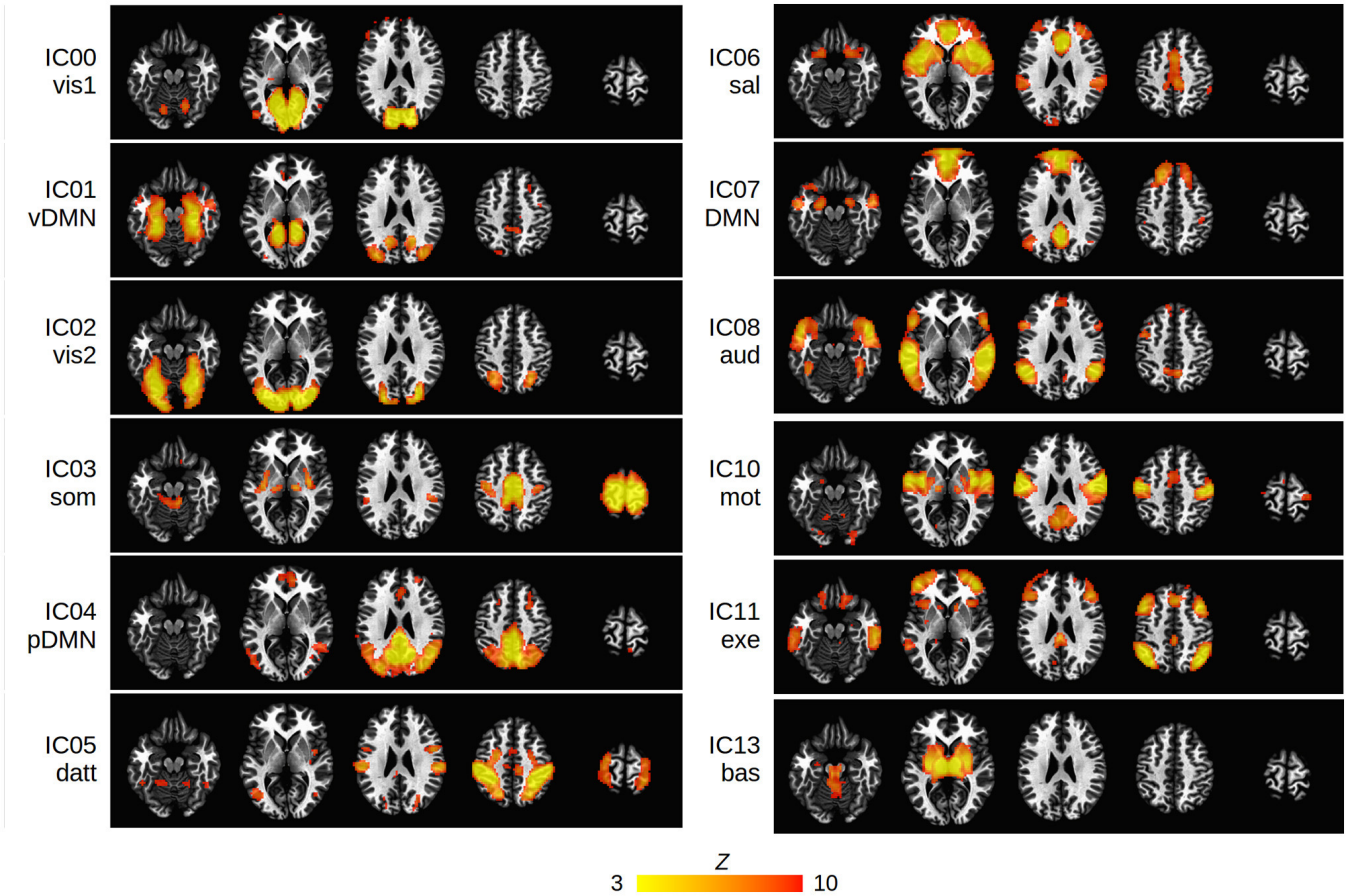
Group ICA maps (thresholded at *Z* > 3) representing the 12 RSNs of interest in the present study: vis1, visual lingual gyrus; vis2, visual occipital lobe; DMN, default mode network; vDMN, ventral DMN; pDMN, posterior DMN; som, somatosensory; datt, dorsal attention; sal, salience; aud, auditory; mot, motor; exe, executive control; bas, basal ganglia. The IC number of each network is shown. Networks are overlayed on standard Talairach-Tournoux (TT) space (left = left).

Twelve cortical and subcortical RSNs of interest were identified using group ICA (Figure 1). The infected and uninfected groups showed no significant FC differences within the ICA-defined RSNs. The 17 spherical seeds that were created at peak FC locations across the 12 RSNs are in Table 2.

**Table 2:** Locations of seeds used in seed-based correlation analyses (SCAs).

Seed region^*^	**Seed network^§^**	**Seed center coordinates (TT, mm)**		
		**x**	**y**	**z**
R cuneus	Visual (vis1)^a^	−1.5	76.5	11.5
R parahippocampal gyrus	vDMN	−25.5	34.5	−6.5
R middle occipital gyrus	Visual (vis2)^a^	−31.5	85.5	5.5
L paracentral lobule	Somatosensory	1.5	28.5	56.5
R cuneus	pDMN	−4.5	67.5	32.5
R cingulate gyrus	pDMN	−4.5	37.5	26.5
R inferior parietal lobule	Dorsal attention	−37.5	37.5	44.5
L precentral gyrus	Dorsal attention	28.5	13.5	56.5
L insula	Salience	37.5	−7.5	2.5
R anterior cingulate	Salience	−1.5	−34.5	14.5
L medial frontal gyrus	DMN	1.5	−61.5	5.5
L cingulate gyrus (posterior portion)	DMN	1.5	46.5	29.5
R superior temporal gyrus	Auditory	−49.5	28.5	2.5
R postcentral gyrus	Motor	−55.5	10.5	17.5
R inferior parietal lobule	Executive control	−40.5	55.5	41.5
R middle frontal gyrus	Executive control	−40.5	−16.5	38.5
R thalamus	Basal ganglia	−7.5	10.5	5.5

**Figure 2:**
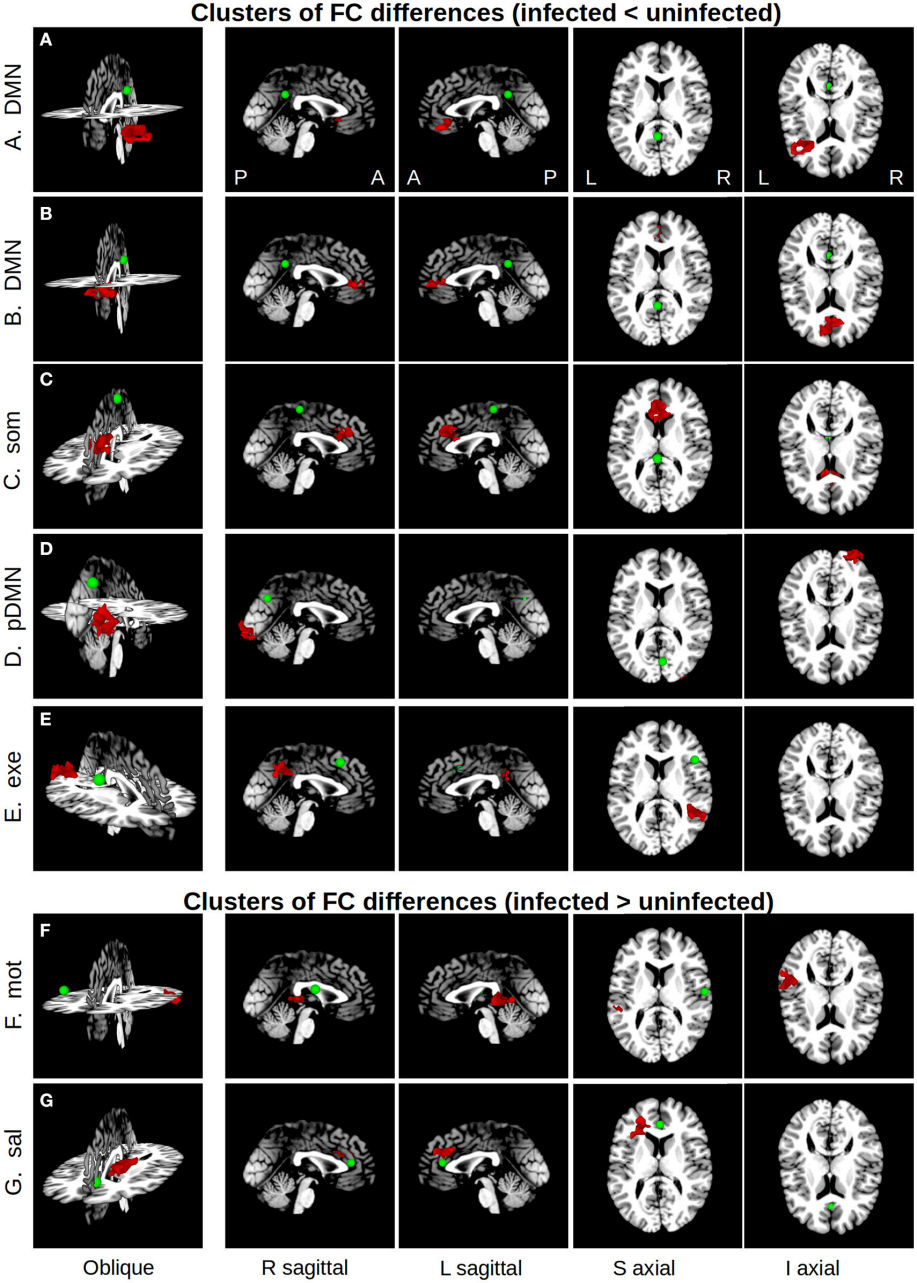
Maps showing clusters of significant FC differences between HIV infected and uninfected groups (red) from SCA analysis, as well as the locations (green spheres, radius = 5 mm). The 3D volume representations were created in SUMA, with sagittal and axial midslices included for reference. From left to right, each column shows: an oblique viewing angle, right and left sagittal views, and superior and inferior axial views. In rows **(A–E)**, the average FC value for the infected group was less than that of the uninfected group, and vice versa in rows **(F,G)**. Table 3 provides seed and cluster location information.

*The spheres (radius = 5 mm) of each seed are shown in Figure 2. Here and below, coordinates are in RAI Dicom standard (“right,” “anterior,” and “inferior” have negative values)*.

* *Based on seed's center in Talairach-Tournoux (TT) atlas*.

§ *Based on components generated by ICA*.

*R, right; L, left; DMN, default mode network; pDMN, posterior DMN; vDMN ventral DMN*.

a *vis1 and vis2 refer to two distinct components of the visual network (visual lingual and visual occipital) generated by ICA*.

Five regions in four WB FC maps showed reduced connectivity to their respective seeds in HIV+ children compared to uninfected controls. The clusters of reduced FC in HIV+ children are shown with their respective seeds in Figure 2, using the 3-dimensional viewer SUMA (Saad et al., 2004; Saad and Reynolds, 2012) within AFNI. Cluster sizes and peak locations are in Table 3, along with overlapping regions in the TT atlas determined using the *whereami* function in AFNI. From a seed in the posterior portion of the left (L) cingulate gyrus in the DMN there were two significant clusters: one overlapping the L inferior frontal gyrus, and another mainly overlapping the L and R anterior cingulate and L medial frontal gyrus. A seed in the L paracentral lobule (somatosensory network) yielded a cluster overlapping the L and R cingulate gyrus and L medial frontal gyrus. A seed in the R cuneus of the posterior DMN exhibited a cluster mainly in the R inferior occipital gyrus, lingual gyrus, and middle occipital gyrus. Finally, a seed in the R middle frontal gyrus (executive control network) resulted in a cluster overlapping the R supramarginal gyrus and inferior parietal lobule.

**Table 3:** Functional connections showing alterations in HIV infected children.

Seed region^*^	**Seed network**	Cluster anatomical location^*^	**Cluster functional network^§^**	**Cluster peak coordinates (TT, mm)**			**Cluster vol (mm^3^)**
				**x**	**y**	**z**	
**CONNECTIONS SHOWING LOWER CONNECTIVITY IN INFECTED CHILDREN**							
L cingulate gyrus (posterior portion)	DMN	L inferior frontal gyrus	Auditory, salience	31.5	−22.5	−3.5	1,728
L cingulate gyrus (posterior portion)	DMN	L AC, R AC, L medial frontal gyrus	DMN	4.5	−46.5	5.5	1,782
L paracentral lobule	Somatosensory	L cingulate gyrus, R cingulate gyrus, L medial frontal gyrus, R AC	Salience, basal ganglia	1.5	−34.5	26.5	2,619
R cuneus	pDMN	R inferior and middle occipital gyrus, R lingual gyrus, R fusiform gyrus	Visual	−16.5	85.5	−6.5	1,566
R middle frontal gyrus	Executive control	R supramarginal gyrus, R inferior parietal lobule	Dorsal DMN, executive control, dorsal attention	−40.5	40.5	26.5	1,620
**CONNECTIONS SHOWING HIGHER CONNECTIVITY IN INFECTED CHILDREN**							
R postcentral gyrus	Motor	L superior temporal gyrus, L middle temporal gyrus	Somatosensory, salience	46.5	31.5	8.5	1,863
R AC	Salience	L medial frontal gyrus, L superior frontal gyrus, L AC	DMN	22.5	−25.5	23.5	1,620

* *Based on seed center and cluster overlap within the Talairach-Tournoux (TT) atlas*.

§ *Based on cluster overlap with the Functional Connectome Project networks (Biswal et al., 2010)*.

*L, left; R, right; AC, anterior cingulate; DMN, default mode network; pDMN, posterior DMN*.

In addition, two regions showed greater FC to their seeds in HIV+ children compared to uninfected controls. These are also shown in Figure 2, with accompanying information in Table 3. A cluster overlapping the L superior and middle temporal gyri showed greater FC in infected children to a seed in the R postcentral gyrus (motor network). A seed in the R anterior cingulate within the salience network resulted in a cluster in the L medial and superior frontal gyri and L anterior cingulate.

**Figure 3:**
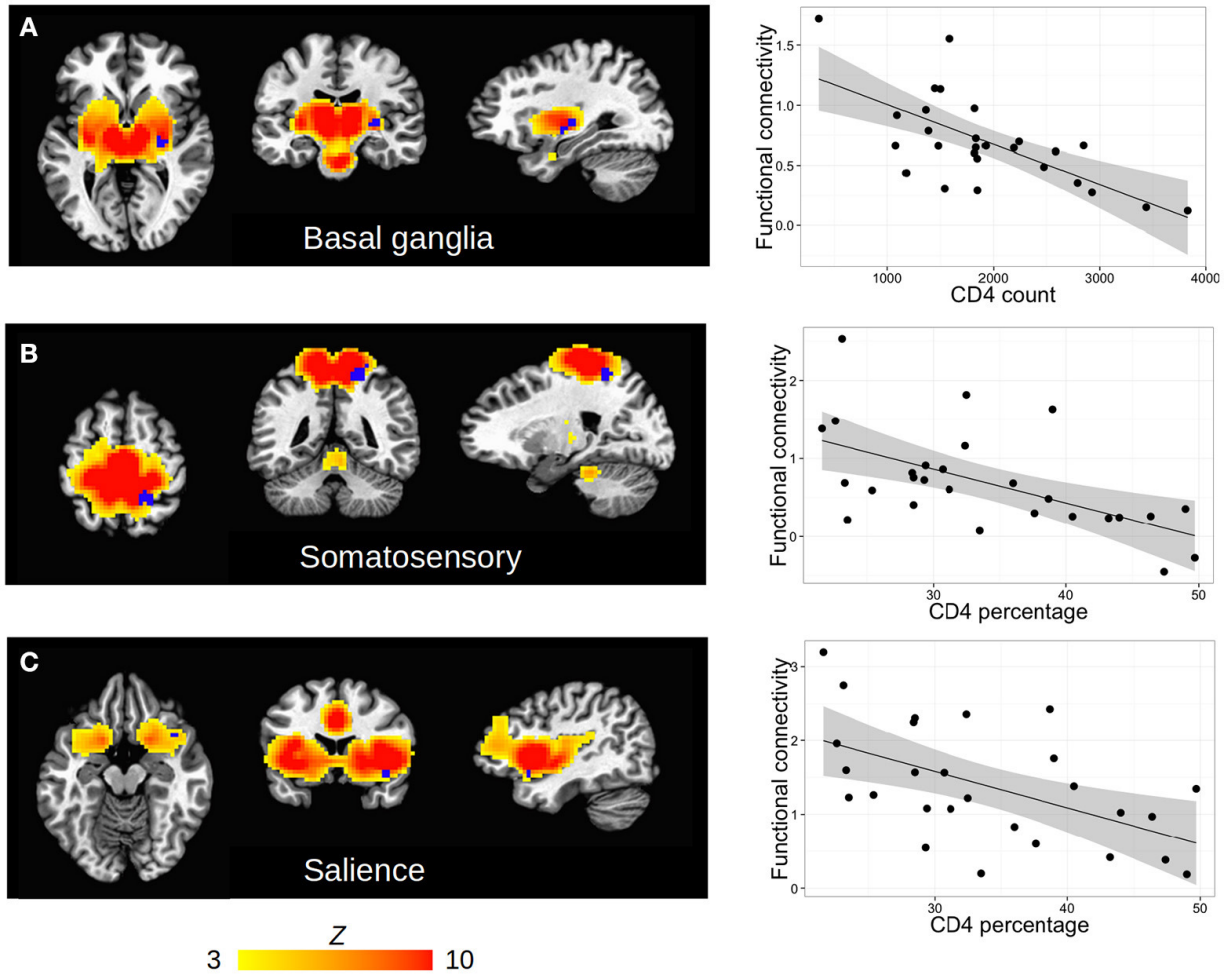
**(A–C)** Clusters (blue) of significant association between FC and clinical measures (either CD4 or CD4%) within the HIV cohort, shown within each RSN defined by group ICA (hot colors, thresholded at *Z* > 3). Slices are shown at the peak coordinates of each cluster, with numbers provided in Table 4. In axial and coronal views, left = left. (Right column) Scatterplots showing each subject's clinical measure vs. mean FC (*Z-*score) within each cluster.

Among HIV+ children, no regions in any RSN showed association of FC with CD4 or CD4% at time of scan. In contrast, poorer immune health in infancy, as reflected by either lower CD4 or CD4% at enrollment (6–8 weeks), was associated with greater FC in three regions in three different RSNs, namely the basal ganglia network (R lentiform nucleus, putamen, and lateral globus pallidus), the somatosensory network (R precuneus, superior parietal lobule, paracentral lobule), and the salience network (R inferior frontal gyrus and insula). The clusters are shown within their respective networks in Figure 3, together with associations of average FC in these clusters with CD4 or CD4%; peak coordinates, location, and volume information are in Table 4.

**Table 4:** Regions within which immunocompromise in infancy (6–8 weeks), defined by low CD4 count and CD4%, is associated with functional connectivity increases at age 7 years.

**ICA-generated network**	Cluster anatomical location^*^	**Cluster peak coordinates (TT, mm)**			**Cluster vol (mm^3^)**	**Cluster mean FC (*Z*)**	**Pearson *r***
		**x**	**y**	**z**			
**CD4 COUNT IN INFANCY ASSOCIATED WITH FC**							
Basal ganglia	R lentiform nucleus, R putamen, R lateral globus pallidus	−28.5	16.5	2.5	513	0.695	−0.672
**CD4% IN INFANCY ASSOCIATED WITH FC**							
Somatosensory	R precuneus, R superior parietal lobule, R paracentral lobule	−19.5	43.5	53.5	918	0.694	−0.579
Salience	R inferior frontal gyrus, R insula	−40.5	−13.5	−12.5	864	1.388	−0.539

* *Based on cluster overlap in Talairach-Tournoux (TT) atlas*.

*R, right; FC, functional connectivity*.

## Discussion

This study investigated HIV-associated FC changes in 7 year old children on two levels: firstly, comparing FC between HIV+ and uninfected cohorts, and secondly, examining relations of FC and HIV clinical measures within the infected group. Contrary to our first hypothesis, we found no group differences between infected and uninfected subjects within the ICA-generated RSNs. However, whole brain SCA from 17 seeds distributed across 12 RSNs revealed 5 connections with lower and 2 with higher connectivity in HIV+ children than controls. Most seeds were in networks previously implicated in HIV (DM, executive control, somatosensory, and salience networks). Of the connections found, all but one (L posterior cingulate to medial prefrontal cortex within DMN) were between networks. Among HIV+ children we observed no association in any of the ICA-generated RSNs with measures of immune health at time of scan. In contrast, poorer immune health in infancy was associated with localized FC *increases* at age 7 years in basal ganglia, somatosensory and salience networks. While we predicted association of FC in somatosensory and salience networks with immune health, the directionality of our findings is opposite to what we hypothesized.

### HIV+ vs. uninfected FC comparisons

The lack of observed HIV-related intra-network differences (i.e., within ICA-generated RSNs) may be due to the developing brain being characterized by less within-network but greater between-network connectivity (Fair et al., 2008; Power et al., 2010; Khundrakpam et al., 2016). It has been postulated that network regions in children are neither isolated fragments of an immature adult system nor unified into cohesive RSNs, but instead integrated into a different network structure organized by anatomical proximity (Fair et al., 2007). Focusing solely upon within-network changes without considering external relationships therefore risks missing critical details about the functional development of RSNs, as well as how specific networks interact with outside brain regions (Power et al., 2010; Khundrakpam et al., 2016) to create the large-scale brain networks essential for efficient functioning (Chen et al., 2008, 2011).

Studies in typically developing healthy children find that long-distance connections between functionally related regions tend to be relatively weak and strengthen with age, while short-distance relationships are stronger and weaken with development (Fair et al., 2009; Power et al., 2010). Synaptic pruning has been proposed as a possible mechanism for reductions in local FC, while myelination throughout childhood and adolescence could facilitate increased long-range correlations (Paus et al., 2001). Here, four of the five connections that demonstrated lower FC in infected children are between frontal and parietal regions, suggesting an HIV-related delay in long-range connection increases. Similarly, greater correlated brain activity in HIV+ children between the AC seed and L medial and superior frontal gyri may result from delay in the age-related decrease of short-range connections. Since decreased prefrontal-parietal connectivity is associated with poorer working memory capacity (Nagy et al., 2004) and performance (Olesen et al., 2004), these developmental delays may have functional consequences requiring further investigation.

While primary sensorimotor connectivity is well established by early childhood (5–8 years), paralimbic connectivity tends to mature in late childhood (8.5–11 years), and connectivity between higher order association regions only in late adolescence (15–18 years) (Khundrakpam et al., 2013). Using interregional correlations in cortical thickness as a measure of structural brain connectivity, Khundrakpam et al. (2013) found that connectivity decreased with age in primary sensorimotor regions but increased in association areas. Greater connectivity in the present study in HIV+ children at 7 years between the R postcentral gyrus in the motor network and L temporal regions in the somatosensory network could therefore reflect a delay in the age-related connectivity reductions between sensorimotor regions.

Using diffusion spectrum imaging, Hagmann et al. (2008) identified a structural core, a single integrated system from which processes in both cortical hemispheres appear to be coordinated, comprising the posterior cingulate cortex (PCC), precuneus, cuneus, paracentral lobule, isthmus of the cingulate, banks of the superior temporal sulcus, and inferior and superior parietal cortices. They further demonstrated that structural and functional connections were strongly correlated, indicating that these regions may similarly be hubs of functional connectivity. It is striking that in the present study all five connections demonstrated FC reductions in HIV+ children involve seeds or clusters located within key components of this core in the posterior medial and parietal cortex. These findings suggest that the structural core may be particularly vulnerable to the effects of HIV and/or ART. Further, since seeds were based on connectivity peaks in our ICA-generated RSNs, our results affirm the important role of these regions in functional integration.

In addition to this mainly posterior medial core, we also observed effects of HIV in medial frontal regions—rostral anterior cingulate (AC) and caudal AC clusters show lower FC to seeds in the PCC and paracentral lobule, respectively, and a seed in the R AC has greater FC to L frontal cortex. In total, four of the six distinct seeds with altered FC in the HIV+ children are medial, and two of these involve connections to medial frontal regions. Neurogenesis during prenatal development occurs in the ventricular zone in the center of the brain, from where neurons migrate radially out to the developing neocortex and connect with other neurons to establish rudimentary neural networks (Stiles and Jernigan, 2010). By the end of the prenatal period, major fiber pathways, including the thalamocortical pathway, are complete. The fact that midline brain regions appear disproportionately affected by HIV suggests that the changes causing the observed HIV-related developmental delays may be occurring early in development.

To our knowledge, only one study previously examined resting state FC in HIV+ youth, in a cohort aged 12–21 years (Herting et al., 2015). Here, WB FC was examined using SCA to 5 seeds within the DMN, but without controls. Functional connections were related to measures of disease severity, specifically peak HIV RNA and nadir CD4%. Greater HIV disease severity was related to both decreases and increases in BOLD signal correlations, and both within- and between networks. Notably, youths with more advanced HIV disease severity showed effects characteristic of a “less mature” DMN, providing additional evidence of HIV-related developmental delay. Internetwork correlations showing effects of disease severity occurred between the DMN seeds and clusters in the executive control, sensorimotor, salience, anterior cingulate/precuneus, and visual networks, with decreased functional connections between the DMN and executive and visual networks being related to worse processing speed scores (Herting et al., 2015). In the present study of 7 year olds, we similarly found HIV-related decreases and increases in FC between the DMN and salience, executive control, and visual networks, as well as lower within-DMN FC. It is noteworthy that many of the same regions are involved in these altered functional connections, specifically the medial prefrontal cortex, PCC, R lateral parietal and occipital cortices, R middle frontal gyrus, L superior frontal gyrus, as well as inferior frontal gyri albeit in different hemispheres. Our results, along with those of Herting et al. point to these regions as being at particular risk of alteration by HIV and/or ART in pediatric populations.

### Functional connectivity associations with clinical measures

In our study, we could not examine associations of FC with peak VL, as done in Herting et al. (2015). In our study peak VLs were truncated at a maximum value of 750,000 copies/mL at baseline. While one might expect timing of worst virological status (peak VL) and poorest immune health (nadir CD4%) to differentially affect FC, due to critical stages of development occurring at different times in childhood in different brain regions and networks, these timings are less meaningful in the context of our cohort where all infected children had either limited ART initiated between 6–12 weeks or deferred treatment when clinically indicated. Notably, Herting et al. (2015) controlled for age of peak VL and nadir CD4% in their analyses. In the CHER cohort where treatment was not based on disease severity but group assignment, nadir CD4% and peak VLs occurred immediately before treatment initiation for most children in whom treatment was deferred, and either at enrollment or after treatment interruption (if interrupted) in children initiating ART before 12 weeks. Therefore, we examined here the influence of immune health on brain development within the HIV+ children by observing the associations between FC and clinical measures at both study enrollment in infancy and time of scanning.

Similar to Thomas et al. (2013), who examined associations of VL and CD4 with FC measures in adults across 5 networks, we also found no regions within any of our ICA-generated RSNs showing a relationship of FC with current CD4 count or CD4%. It is possible that SCA, which assesses also between-network connectivity, could be more sensitive to detect connections affected by current immune health at this age. In contrast, poorer immune health in infancy was associated with increased FC in three right-lateralized regions in separate RSNs—basal ganglia, somatosensory, and salience networks. These findings imply that infant immune health has long-term consequences on brain development.

An MR spectroscopy study by Mbugua et al. (2016) of 5-year-old children from the same cohort similarly found that immune health measures at 6–8 weeks were related to N-acetyl aspartate (NAA) and choline levels in the basal ganglia, despite early ART, and VL suppression. The metabolite NAA is associated with neuronal density and integrity, and the result suggests that poor immune health in infancy relates to basal ganglia neuronal populations at age 5. If early HIV infection impacts basal ganglia neuronal density or integrity, neuronal activity and therefore FC in the region may be altered; however additional work is needed to directly examine possible relationships between altered FC and metabolite levels within this cohort. Notably, the basal ganglia are one of the mostly widely reported HIV-affected regions of the brain across modalities (e.g., Berger and Arendt, 2000; Moore et al., 2006; Ellis et al., 2007; Gongvatana et al., 2013).

Synaptogenesis and synaptic pruning start around 20 weeks gestational age (GA), and myelination around GA 30–32 weeks (Casey et al., 2005). These processes start in primary sensorimotor regions and sensory tracts, progressing to parietal and temporal association cortex, and finally prefrontal cortex (Khundrakpam et al., 2016). Correlated brain activity has been demonstrated in premature infants from 30 weeks GA, including in the somatosensory, visual, auditory, pDMN, and salience networks (Kiviniemi et al., 2000; Fransson et al., 2007; Redcay et al., 2007; Lin et al., 2008; Smyser et al., 2010). It is worth noting that the three networks where we found effects of immune health in infancy on RSFC are all involved in primary motor and sensory functions. The somatosensory network processes peripheral inputs and tactile sensations and is important for controlling action (Lin et al., 1996). The salience network, comprising the dorsal AC, the left and anterior right insula, and the adjacent inferior frontal gyri (Seeley et al., 2007), is important in coordinating behavioral responses (Medford and Critchley, 2010), initiating cognitive control (Menon and Uddin, 2010), and maintaining and implementing task sets (Dosenbach et al., 2006; Nelson et al., 2008). The basal ganglia network, which includes the putamen and caudate bilaterally as well as anterior parts of the thalamus (Szewczyk-Krolikowski et al., 2014), primarily regulates motor control, but also plays a role in human reasoning and adaptive function, the control of reward-based learning, sequencing, and cognitive function (Leisman et al., 2014). Since these networks support functional domains that are crucial when an infant starts to interact with his/her environment, they are amongst the first to mature and may be more sensitive to poor immune health during critical stages of development in infancy. However, it remains unclear why resulting FC would be *increased* at the observed stage of childhood. Connectivity increases with greater HIV disease severity were also observed by Herting et al. (2015) in youth between the R inferior temporal cortex within the DMN and the brainstem, R middle frontal gyrus (anterior cingulate/precuneus network), and R frontal pole (salience), and between the executive control and salience networks in HIV+ adults compared to uninfected controls (Thomas et al., 2013). In contrast to our finding of hyperconnectivity *within* networks in the children with the poorest immune health in infancy, the connectivity increases reported by the two other studies were *between* networks, indicating less independent brain networks in infected individuals, consistent with impairment. It is not clear whether the within-network FC increases observed here reflect an advantage or a deficit.

Since connectivity within local networks decreases with age from as young as 4–9 months (Damaraju et al., 2014), the FC increases observed at age 7 years in children with poorer immune health in infancy could be due to decreased synaptic pruning. The immune system plays a critical role in normal brain development and following injury (Merrill, 1992; Zhao and Schwartz, 1998; Hanamsagar and Bilbo, 2016), and elevated levels of cytokines and their receptors from perinatal infection have been linked with abnormal brain development and an increased risk of neurodevelopmental disorders (Urakubo et al., 2001; Pang et al., 2003; Meyer et al., 2006). The morphology and function of microglia, the primary immune cells in the brain, shift from an immature to a mature state throughout brain development in an age- and brain region-dependent manner (Bilbo, 2013). Animal models have shown that a single neonatal infection alters microglial functioning, leading to exaggerated cytokine production within the brain in response to subsequent immune challenges and an increased risk of cognitive deficits later in life (Bilbo, 2013). Since microglia are long-lived, functionally altered microglia may remain in the brain into adulthood. Among their many roles, microglia aid in synaptic pruning and regulate synaptic plasticity and function (Schafer et al., 2012; Hong et al., 2016). Following localization of C1q, the initiating protein within the classical complement cascade of the immune system, to synapses within the postnatal brain intended for elimination, microglia expressing the complement receptor for this protein are activated (Stevens et al., 2007; Schafer et al., 2012). We hypothesize that changes in the developmental trajectory of microglia arising from perinatal HIV infection and neuroinflammation in infancy alters later-life immune function, causing disruptions in synaptic pruning and connectivity increases within affected networks in childhood.

Given the overlapping functionality of the three affected networks, our findings provide impetus for further investigation of FC with motor and sensory performance measures. Such analysis may provide insight into whether the observed FC increases impact children positively, in the form of a possible compensatory mechanism, or negatively, such as delayed or impaired synaptic pruning, at this age.

### Limitations

Here, we used a voxelwise threshold of *p* = 0.005 during the clustering procedure. We note that more conservative voxelwise thresholding at *p* = 0.001 produced no significant results, likely due to the small sample size in this study. However, voxelwise thresholding with *p* = 0.005 showed adequate familywise error rate control when using the mixed ACF modeling (Cox et al., 2017) implemented here. In addition, because all HIV+ children were on ART it is impossible to disentangle the contributions of HIV infection and ART to our findings. Lastly, in these children we do not know whether HIV infection occurred prenatally or during birth. This knowledge would allow us to better understand the timing of damage during the fetal period and exposure to other viruses or bacteria which may have primed the immune system.

## Conclusion

HIV infection in conjunction with early ART alters between-network connectivity in children (here, measured at age 7 years). The predominance of medial brain regions suggest that HIV affects brain development from its earliest stages. The networks implicated include DMN, somatosensory, salience, motor, basal ganglia, visual and auditory, as well as the higher-order executive control network. Weaker long-distance and stronger short-range connections in HIV+ children suggest developmental delay. Further, although no associations were found with current immune health, poor immune health during infancy is associated with localized FC increases in somatosensory, salience, and basal ganglia networks, indicating that effects of immunocompromise during critical stages of development in early infancy persist into childhood, despite early ART and viral suppression. These neurobiological alterations may contribute to cognitive problems among HIV infected children (e.g., Lewis-de los Angeles et al., 2017; Yadav et al., 2017) and require further investigation.

## Author contributions

EM, BL, and AvdK were involved in the study design and acquisition of data. JT, MH, PT, FL, SG, and BB were involved in data and statistical analyses. JT, EM, PT, and MH drafted the work and all other authors provided critical revision of the manuscript. JT, PT, MH, EM, BL, ED, and MC provided interpretation of data.

### Conflict of interest statement

The authors declare that the research was conducted in the absence of any commercial or financial relationships that could be construed as a potential conflict of interest.

## Appendix A

The afni_proc.py command within AFNI was used to specify all steps (or “blocks”) and options for the full processing pipeline in this study. The command-based configuration provides a succinct form for generating a flexible analysis pipeline, and, unlike a GUI-generated analysis, it both ensures that identical steps are carried out across subjects and maintains an exact record of all steps for reproducibility. The afni_proc.py command used in the present study is presented in Table A1, and the summary of steps is provided in the section Methods.

**Table A1:** The afni_proc.py command using in AFNI (Cox, 1996).

afni_proc.py	\
-subj_id $sub_name	\
-dsets $rest_set	\
-copy_anat $anat_set	\
-blocks despike tshift align tlrc volreg blur mask regress	\
-tcat_remove_first_trs 5	\
-tlrc_NL_warp	\
-tlrc_base ~/abin/TT_N27+tlrc	\
-volreg_tlrc_warp	\
-blur_size 6.0	\
-mask_apply epi	\
-mask_segment_anat yes	\
-regress_bandpass 0.01 0.1	\
-regress_apply_mot_types demean deriv	\
-regress_ROI WMe CSFe	\
-regress_RSFC	\
-regress_run_clustsim no	\
-regress_est_blur_errts	

The first three variables ($sub_name, $rest_set and $anat_set) are respectively set for each subject's ID, resting state EPI data set and anatomical volume.
